# Genome-Based Evaluation of Safety and Probiotic Traits in Infant Feces-Sourced *Bifidobacterium animalis* subsp. *lactis* BD1

**DOI:** 10.3390/foods15020316

**Published:** 2026-01-15

**Authors:** Meng Tian, Zihao Liu, Jiahang Li, Jialin Wang, Dayong Ren, Yue Leng

**Affiliations:** College of Food Science and Engineering, Jilin Agricultural University, Changchun 130118, China; jodie0730@163.com (M.T.); m17631616248@163.com (Z.L.); l1719541831@163.com (J.L.); 13803454487@163.com (J.W.)

**Keywords:** *Bifidobacterium animalis* subsp. *lactis* BD1, whole-genome sequencing, safety assessment, antibiotic resistance, probiotic properties

## Abstract

*Bifidobacterium animalis* subsp. *lactis* is a widely used probiotic, yet its efficacy is highly strain-specific, and growing antibiotic resistance necessitates rigorous safety evaluations. We used whole-genome sequencing and in vitro assays to characterize the safety and probiotic traits of infant feces-sourced strain BD1, which shows preliminary mood-modulating and anti-inflammatory potential. The BD1 genome showed a favorable safety profile. VFDB analysis identified 139 low-similarity homologs, with no major toxins detected. Only four chromosomally encoded antibiotic resistance genes were found; phenotypic testing confirmed resistance solely to tetracycline and mupirocin. Although the tetracycline resistance gene *tet*(*W*) was identified in genomic island GI01, the absence of associated mobile genetic elements results in a negligible risk of its mobilization. Functional annotation highlighted a dominant metabolic capacity for carbohydrate and amino acid metabolism. BD1 is rich in CAZymes, enabling superior utilization of diverse substrates (starch, sucrose, galactose). Enrichment in lipid metabolism pathways (glycerolipid, sphingolipid) further suggests potential for enhancing fermented product flavor. In vitro assessment demonstrated moderate gastrointestinal tolerance and strong bile salt tolerance. Surface properties showed pronounced cell surface hydrophobicity and confirmed biofilm-forming potential. In conclusion, BD1 exhibits robust safety, metabolic versatility, and strong probiotic characteristics, supporting its development as a functional probiotic strain.

## 1. Introduction

*Bifidobacterium* comprises Gram-positive, pleomorphic, and strictly anaerobic bacteria that represent a major component of the intestinal microbiota in humans and other warm-blooded animals [1]. Owing to its excellent fermentation characteristics and beneficial effects on human health, it is widely used in the food and pharmaceutical industry [2,3]. Commonly employed strains include *Bifidobacterium longum* subsp. *longum*, *Bifidobacterium breve*, *Bifidobacterium animalis* subsp. *lactis*, and *Bifidobacterium adolescentis*, among others. Among these, *Bifidobacterium animalis* subsp. *lactis* is one of the most extensively studied and widely applied species. It is commonly used in a range of probiotic supplements, infant formula, and fermented dairy products. Furthermore, it has been shown to confer multiple beneficial effects within the host intestine, including the potential to enhance cognitive function and mitigate gastrointestinal disorders [4,5,6,7]. However, current research has revealed variations in therapeutic efficacy and fermentation performance among different strains [8,9]. Due to the strain-specific characteristics, many *Bifidobacterium animalis* subsp. *lactis* fail to meet expectations as nutritional supplements or starter cultures. Consequently, there is a growing need to identify and develop more effective probiotic strains. Previously, we isolated a strain of *Bifidobacterium animalis* subsp. *lactis* BD1 from infant feces. Preliminary assessment indicates its potential to improve host mood and reduce inflammation levels.

Although *Bifidobacterium animalis* subsp. *lactis* is generally recognized as safe based on traditional knowledge, its increasing antibiotic resistance poses a growing health concern [10]. Therefore, a detailed analysis of the BD1 strain is essential to evaluate its potential for further application in the food and pharmaceutical industries. Previous studies have primarily assessed the safety and characteristics of probiotics through in vitro phenotypic assays, often lacking mechanistic insights at the genetic level. Recently, whole-genome sequencing has emerged as a powerful tool for comprehensive microbial characterization [11]. This approach employs high-throughput technologies to obtain complete genome sequences, enabling in-depth analysis of functional and structural gene features. Furthermore, genomic analysis will provide deeper insights into the beneficial properties of the BD1 strain at the genetic level, thereby contributing to a more comprehensive understanding of its mechanisms underlying mood modulation and anti-inflammatory effects. By validating the safety and probiotic properties of the BD1 strain, this study not only expands the resources of probiotic lactic acid bacteria in China, but also provides a foundation for its broader application in functional foods and pharmaceutical formulations.

In our previous study, a strain of *Bifidobacterium animalis* subsp. *lactis*, designated BD1, was isolated from infant fecal samples. This strain demonstrated efficacy in alleviating depression and inflammation in preliminary investigations (The data to be published). To assess its safety for potential applications, we conducted a comprehensive characterization of its probiotic properties and safety profile using a combination of whole-genome sequencing and in vitro experiments. These results provide a robust scientific foundation for further development and utilization of the functional probiotic strain *Bifidobacterium animalis* subsp. *lactis* BD1.

## 2. Materials and Methods

### 2.1. Growth Conditions of the Bacterial Strains

The experimental strain BD1, originally isolated from healthy infant feces, is deposited in the Guangdong Microbial Culture Collection Center (GDMCC) under the accession number GDMCC No. 65985. For the present study, the strain was cultured anaerobically at 37 °C for 48 h in de Man–Rogosa–Sharpe (MRS) medium supplemented with 0.05% L-cysteine.

### 2.2. Genome Sequencing

#### 2.2.1. Genomic DNA Extraction

The BD1 strain was first streaked onto MRS agar and incubated at 37 °C for 12 h. A single colony was then transferred into 200 mL of MRS broth and cultured under anaerobic conditions at 37 °C with shaking at 150 rpm for approximately 12 h. Bacterial cells were harvested by centrifugation at 12,000× *g* for 10 min. Genomic DNA was extracted using a Bacterial DNA Extraction Kit (Majorbio, Shanghai, China) following the manufacturer’s instructions. The purified DNA was quantified, and high-quality samples were retained for subsequent analysis.

#### 2.2.2. Library Construction and Genome Sequencing

Genomic DNA was sequenced using both the PacBio Sequel IIe (Pacific Biosciences, Menlo Park, CA, USA) and Illumina platforms. For Illumina sequencing, standard library construction was performed as follows. Genomic DNA was sheared into 400–500 bp fragments using a Covaris M220 Focused Acoustic Shearer (Covaris, Woburn, MA, USA). Libraries were then prepared using the NEXTFLEX Rapid DNA-Seq Kit (Bioo Scientific, Austin, TX, USA). The procedure involved end-repair and phosphorylation of the DNA fragments, followed by A-tailing and adapter ligation. The adapter-ligated products were subsequently enriched by PCR. The final libraries were sequenced on an Illumina NovaSeq 6000 platform (Illumina Inc., San Diego, CA, USA) to generate 2 × 150 bp paired-end reads.

For PacBio sequencing, genomic DNA was sheared to ~10 kb, and the fragments were subjected to purification, end-repair, and ligation with SMRTbell sequencing adapters following the manufacturer’s recommendations (Pacific Biosciences, CA, USA). The final library was sequenced on a single SMRT cell using the standard protocol.

#### 2.2.3. Genome Assembly and Annotation

The data generated from PacBio Sequel IIe and Illumina platform were used for bioinformatics analysis. All of the analyses were performed using the online platform of Majorbio Cloud Platform (http://cloud.majorbio.com) from Shanghai Majorbio Bio-pharm Technology Co., Ltd. (Shanghai, China). The detailed procedures are as follows.

The raw Illumina sequencing reads generated from the paired-end library were subjected to quality-filtered using fastp v0.23.0. The HiFi reads generated from the PacBio platform for analysis. Then the clean short reads and HiFi reads were assembled to construct complete genomes using Unicycle v0.4.8 and uses Pilon v1.22 to polish the assembly using short-read alignments, reducing the rate of small errors [12]. The coding sequences (CDs) of chromosome and plasmid were predicted using Prodigal v2.6.3 and GeneMarkS respectively [13,14]. tRNA-scan-SE (v 2.0) was used for tRNA prediction and Barrnap v0.9 (https://github.com/tseemann/barrnap (accessed on 12 December 2025)) was used for rRNA prediction [15]. The predicted CDs were annotated from NR, Swiss-Prot, Pfam, GO, COG, KEGG and CAZY database using sequence alignment tools such as BLAST v2.3.0, Diamond v0.8.35 and HMMER v3.1b2. Briefly, each set of query proteins were aligned with the databases, and annotations of best-matched subjects were obtained for gene annotation.

### 2.3. Antibiotic Resistance Test

Antibiotic MICs were determined by the broth microdilution method [16]. Two-fold serial dilutions of antibiotics (0.5–4096 mg/L) were prepared in a microplate. Bacterial strains were diluted to 5 × 10^5^ CFU/mL in MH broth and inoculated into the wells. After 24 h of incubation at 30 °C, the MIC was recorded as the lowest concentration preventing visible growth. Breakpoints were set based on the MIC distribution within the population, with isolates exceeding this value deemed resistant. All assays were performed in three independent replicates.

### 2.4. Cell Surface Hydrophobicity

Cell surface hydrophobicity of BD1 was evaluated using the bacterial adhesion to xylene (BATH) assay, adapted from a standard protocol. After growing overnight in MRS broth at 37 °C, cells were harvested, washed, and resuspended in PBS (pH 7.0) to an OD600 of 0.5 ± 0.05. An equal volume of xylene was added, and the mixture was vortexed for 5 min. The OD600 of the aqueous phase was measured after 15, 30, and 60 min of phase separation at room temperature. Hydrophobicity was calculated as follows:

$$Hydrophobicity\;\%=\frac{P_{0}-P_{1}}{P_{0}}\times 100$$

where *P*_0_ and *P*_1_ are the absorbance values before and after extraction with hydrocarbons, respectively.

### 2.5. Evaluation of Auto-Aggregation

The auto-aggregation potential of BD1 was evaluated following a published protocol [17]. Cell suspensions were prepared and standardized to an OD600 of 0.5 ± 0.05. After initializing static incubation at 37 °C to simulate growth conditions, the optical density at 600 nm was recorded from the upper phase at 4, 8, 12, 16, 20, and 24 h, respectively. The auto-aggregation rate was determined using the formula below:

$$Auto-aggregation\;\%=1-\left(\frac{P_{1}}{P_{0}}\right)\times 100$$

where *P*_0_ is the absorbance at time 0, and *P*_1_ is the absorbance detected after 4, 8, 12, 16, 20, and 24 h.

### 2.6. Effect of Gastric and Intestinal Juice on the Viability of BD1

The gastrointestinal tolerance of BD1 was analyzed following a modified protocol from Lu et al. (2024) [10]. Briefly, simulated gastric juice was composed of 2 g/L NaCl and 3.2 g/L pepsin, with pH adjusted to 2.0 using 5 M HCl. Simulated intestinal juice contained 6.8 g/L KH_2_PO_4_ and 10 g/L pancreatin, with pH adjusted to 8.0 using 0.1 M NaOH. Bacterial cells were harvested from an overnight culture via centrifugation (5000× *g*, 15 min, 4 °C), washed, and resuspended in PBS. These suspensions were then mixed with PBS (positive control), gastric juice, or intestinal juice to standardize the initial OD600 to 1.0. Bacterial viability was determined at 37 °C after varying periods of tolerance.

### 2.7. Bile Salt Tolerance of BD1

The bile salt tolerance of the strain was evaluated by growing it in MRS broth supplemented with 0, 0.1, 0.3, and 0.5% (*w*/*v*) bile salts. The bacterial suspension was inoculated at a 1:10 ratio into the medium and incubated at 37 °C. Viable cell counts were determined at 0 and 4 h post-inoculation to assess survival.

### 2.8. Biofilm Formation Ability of BD1

The capacity of strain BD1 to form biofilms was analyzed according to established methods with minor modifications [18]. Bacterial suspensions were standardized to an OD600 of 0.2 in fresh MRS broth and aliquoted into 96-well plates, followed by static incubation at 37 °C for 72 h. Following adhesion, the wells were rinsed twice with PBS to remove planktonic cells. The remaining adherent biomass was stained with crystal violet for 20 min, and unbound dye was removed by subsequent PBS washes. Biofilm structures were visualized under an inverted microscope at 40× magnification, and image analysis was performed using the 3D surface plot module of ImageJ software (version 1.52i).

### 2.9. Statistical Analysis

All data are presented as the mean ± standard deviation (SD) from three independent experiments, each performed in duplicate. Statistical analyses were performed using one-way analysis of variance (ANOVA) followed by Tukey’s post hoc test for multiple comparisons, with GraphPad Prism software (version 8.0). Differences were considered statistically significant at * *p* < 0.05, with ** *p* < 0.01 and *** *p* < 0.001 representing higher degrees of significance.

## 3. Results

### 3.1. Genomic Characteristics of B. animalis BD1

**Figure 1:**
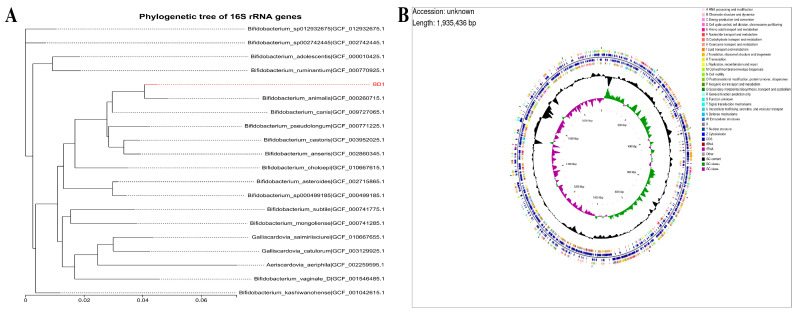
(**A**) Phylogenetic tree based on 16S rRNA gene sequences. (**B**) General features of the BD1 genome.

Based on gene sequence similarity, the constructed phylogenetic tree demonstrates a close evolutionary relationship between strain BD1 and *Bifidobacterium animalis* GCF_000260715.1 (Figure 1A). This phylogenetic placement is consistent with the observed similarities in their physiological and biochemical profiles. The genome of strain BD1 consists of a single circular chromosome spanning 1,935,436 bp, with a G+C content of 60.49% (Table 1; Figure 1B). A total of 1558 genes were annotated, with an average length of 1079.49 bp, collectively representing 86.90% of the genome. Prediction of non-coding RNAs using tRNAscan-SE and barrnap v0.9 identified 52 tRNA genes, 9 rRNA genes (comprising three copies each of the 5S, 16S, and 23S rRNA), and 9 sRNA genes. The quality of the whole-genome sequencing was assessed based on genome coverage depth, contig distribution, and assembly completeness, as presented in Supplementary Figure S1 and Tables S1–S3. These metrics confirm the reliability of our sequencing data and the downstream analyses.

**Figure 2:**
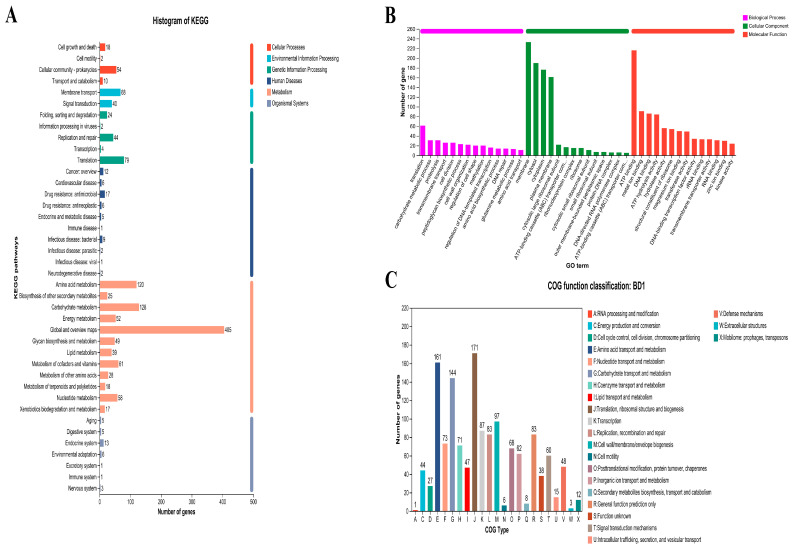
(**A**–**C**) Functional annotation of the BD1 genome. ((**A**): KEGG; (**B**): GO; (**C**): COG).

Functional annotation using the KEGG database assigned 1024 genes from the BD1 genome to various metabolic pathways (Figure 2A). The distribution revealed a high abundance of genes involved in metabolic processes (948 genes), with substantial contributions from genetic information processing (153 genes), environmental information processing (108 genes), and cellular processes (84 genes). Genes related to human diseases and organismal systems were less prevalent. Among the metabolic subcategories, carbohydrate and amino acid metabolism were the most predominant, indicating a highly active metabolic profile for these compounds. A total of 1313 coding genes in the BD1 genome were categorized by Gene Ontology (GO), with 869, 1102, and 800 genes associated with cellular components, molecular functions, and biological processes, respectively (Figure 2B). Moreover, COG analysis classified 1290 protein-coding genes from the BD1 genome into functional categories, with the most abundant groups being translation, ribosomal structure and biogenesis (171 genes), amino acid transport and metabolism (161 genes), and carbohydrate transport and metabolism (144 genes) (Figure 2C). A key finding from the functional annotation is the convergence between the COG and KEGG databases, both highlighting a dominant representation of genes involved in carbohydrate and amino acid metabolism. This strongly suggests that these pathways constitute fundamental metabolic capabilities of strain BD1.

### 3.2. Analysis of Virulence Factors (VFDB) of BD1

Analysis via the Virulence Factor Database (VFDB) identified 139 genes in the BD1 genome as putative virulence factor homologs. However, the majority of these genes exhibited limited similarity (<50%) to established virulence factors in the database, suggesting that BD1 may not harbor functionally conserved virulence determinants. In addition, these genes have not been previously reported to be related to the pathogenicity of B. animalis. In addition, comprehensive genome annotation revealed the absence of genes encoding major known toxins, including hemolysin BL (*Hbl*), non-hemolytic enterotoxin (*Nhe*), cytotoxin K, cereulide (*Ces*), and biogenic amine-producing enzymes.

### 3.3. Analysis of Antibiotic Resistance of BD1

Genomic analysis identified four putative antibiotic resistance genes in the BD1 chromosome, conferring potential resistance to tetracycline, aminoglycosides, mupirocin, and rifamycin (Table S4). Since no plasmid was detected, these genes are chromosomally encoded, which minimizes the risk of horizontal transfer. To validate these predictions, phenotypic susceptibility testing was performed using the broth microdilution method. The results confirmed resistance only to tetracycline and mupirocin, for which the minimum inhibitory concentrations (MICs) were determined (Table 2).

### 3.4. Gene Island Analysis of BD1

**Figure 3:**
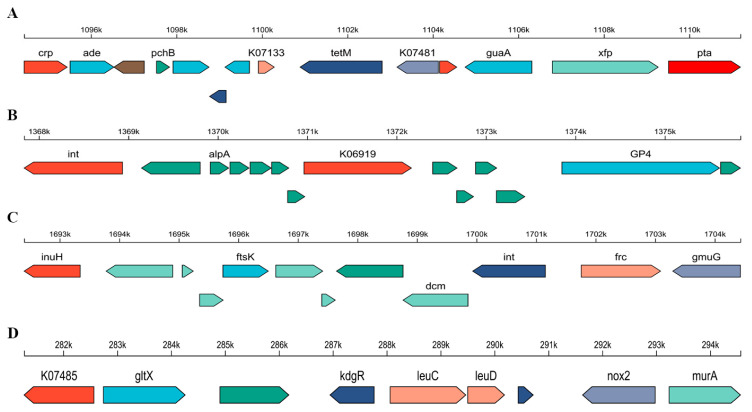
Linear maps of the genomic islands identified in the BD1 genome. (**A**) GI01, (**B**) GI02, (**C**) GI03, (**D**) GI04.

Genomic islands (GIs) are horizontally acquired DNA segments in bacterial genomes that often carry functionally adaptive genes, such as those involved in antibiotic resistance or specialized metabolic pathways [19]. These mobile elements play a crucial role in bacterial evolution by facilitating rapid environmental adaptation. In the genome of strain BD1, four major genomic islands were identified, each enriched in genes likely acquired through horizontal gene transfer, collectively contributing to the strain’s environmental adaptability and potential functional diversification. Among these, GI01 was the most notable, harboring a variety of genes including the tetracycline resistance gene *tet*(*W*), along with several metabolism- and regulation-related genes annotated as “MULTISPECIES”, suggesting a possible horizontal origin (Figure 3A). GI02 exhibited typical prophage-like features, encoding a site-specific integrase, phage protease, and numerous hypothetical proteins, indicative of historical phage integration (Figure 3B). However, no prophage region was detected in BD1 using Phage_Finderv2.4.0, suggesting a potential false-positive prediction in the GI annotation for this region. GI03 and GI04 were primarily enriched with metabolic genes. GI03 contained genes encoding formyl-CoA transferase and cellulase, while GI04 was associated with amino acid metabolism and cell wall synthesis (Figure 3C,D). These genes likely expand the metabolic versatility of BD1. In summary, these genomic islands collectively endow the strain with antibiotic resistance, metabolic flexibility, and genomic plasticity, which may be critical for its survival and evolution in complex ecological niches.

### 3.5. Fermentation Properties of BD1

#### 3.5.1. Carbohydrate Metabolism

**Figure 4:**
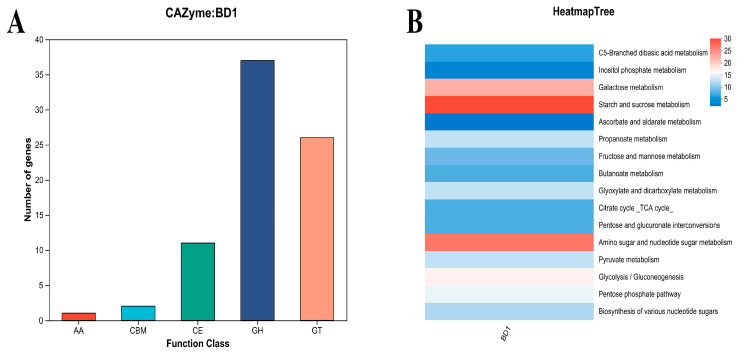
(**A**) Carbohydrate-active enzymes (CAZymes) identified from the whole genome. (**B**) KEGG pathways enriched in carbohydrate metabolism.

Carbohydrate-active enzymes (CAZymes) are a major class of multifunctional enzymes that play a central role in fermentation by dictating the conversion of substrates into key metabolites. Figure 4A shows that the genome of BD1 is predominantly enriched with genes encoding glycosidases and glycosyltransferases. This genetic repertoire equips it with the capability to precisely modulate the sugar metabolism network, which in turn influences flavor formation, the synthesis of functional components, and overall fermentation efficiency. Moreover, KEGG annotation results further reveal significant enrichment of metabolic pathways in BD1, particularly in starch and sucrose metabolism, amino sugar and nucleotide sugar metabolism, and galactose metabolism (Figure 4B). This genetic profile suggests a superior capacity to utilize a wide range of substrates, including starchy grains, sugary fruits, and galactose-containing materials such as whey and legumes. Furthermore, the interaction of BD1’s metabolic network produces metabolites such as organic acids and esters, thereby exerting a significant influence on final product quality by regulating flavor and improving texture (Figure S2). These attributes demonstrate that BD1 not only serves as a robust fermentation starter but also acts as a powerful biocatalyst for tailoring the sensory attributes of fermented products.

#### 3.5.2. Lipid Metabolism

**Figure 5:**
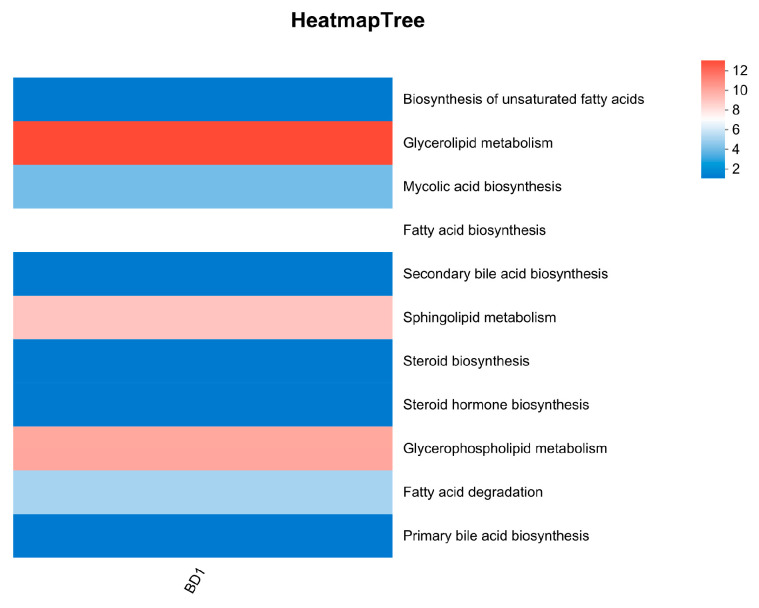
KEGG pathways enriched in lipid metabolism.

The ability of a starter strain to mediate lipid metabolism—regulating the synthesis, breakdown, and transformation of lipids—can significantly enhance the nutritional value and functional properties of fermented products. Therefore, the presence of robust lipid metabolism should be considered a critical criterion in the selection of excellent starter cultures. Genomic annotation identified significant enrichment of lipid metabolism genes in BD1 within the glycerolipid, glycerophospholipid, and sphingolipid pathways, highlighting its strong potential for modulating diverse lipid components (Figure 5 and Figure S3). This indicates that BD1 can enhance aroma complexity in fermented meat products through coordinated lipid metabolism. Specifically, glycerolipid metabolism promotes lipid hydrolysis to release free fatty acids, which are precursors for volatile esters and ketones. Concurrently, sphingolipid metabolism contributes to the accumulation of additional flavor precursors. These parallel processes collectively enrich the flavor profile of lipid-rich substrates.

#### 3.5.3. Amino Acid Metabolism

**Figure 6:**
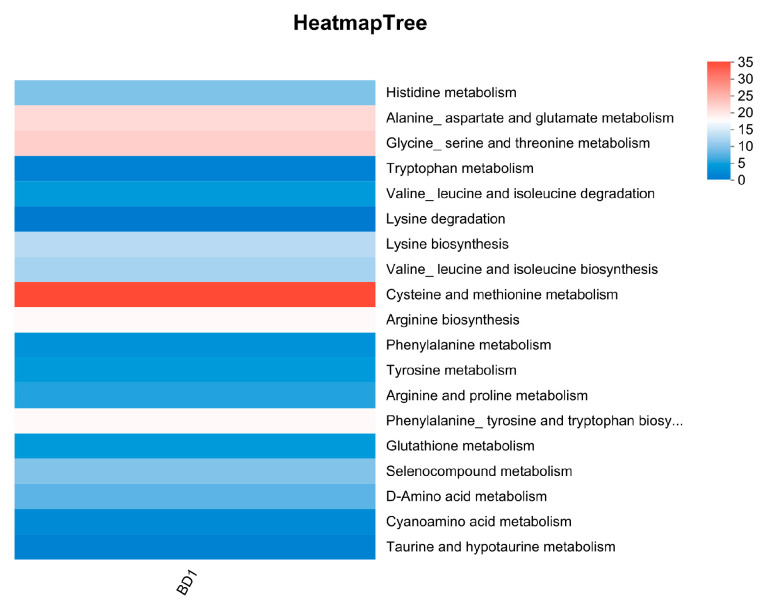
KEGG pathways enriched in amino acid metabolism.

Amino acid metabolism plays a central role in fermentation, as it governs critical processes in product synthesis and flavor development. As such, the metabolic genes in a strain dictate the transformation of amino acids, ultimately determining key attributes including fermentation efficiency, nutritional value, and flavor complexity. KEGG annotation identified 199 genes in BD1 involved in amino acid metabolism and Metabolism of other amino acids, with significant enrichment in cysteine and methionine metabolism, glycine, serine and threonine metabolism, and alanine, aspartate and glutamate metabolism (Figure 6). The enzymatic conversion of these amino acids, directed by the corresponding genes, yields metabolites such as sulfur-containing compounds and γ-aminobutyric acid, which contribute significantly to the unique flavor profiles of various fermented foods, including cheese, fermented meat, and bean products (Figure S4).

### 3.6. Assessment of the Probiotic Properties of BD1

#### 3.6.1. Tolerance Analysis of BD1

**Figure 7:**
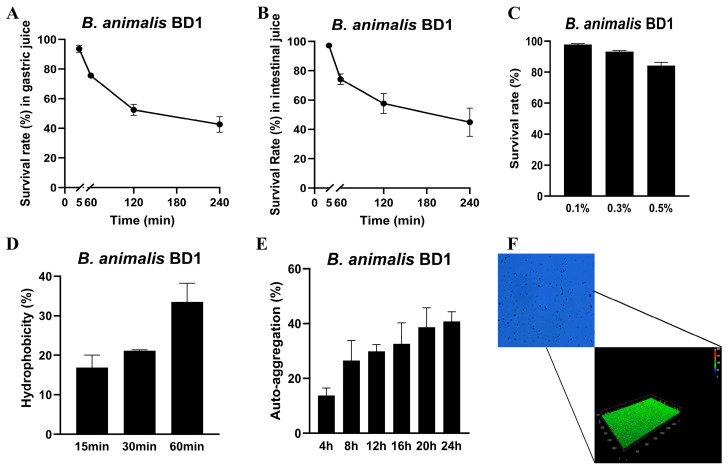
(**A**,**B**) Tolerance of simulated gastric juice (**A**) and simulate intestinal juice (**B**) with BD1. (**C**) Tolerance of bile salt with BD1. (**D**) Cell surface hydrophobicity of BD1. (**E**) Auto-aggregation ability of BD1. (**F**) Biofilm formation ability of BD1.

The in vitro gastrointestinal tolerance of strain BD1 was evaluated by determining its survival rate in simulated gastric and intestinal juices. After 240 min of exposure, the survival rates were 42.59% in gastric juice (Figure 7A) and 44.86% in intestinal juice (Figure 7B), demonstrating a moderate tolerance to both conditions. In addition, BD1 also exhibited significant bile salt tolerance, as evidenced by survival rates greater than 80% across all tested concentrations (0.1–0.5%) (Figure 7C). These results are consistent with a robust innate capacity to withstand the biliary environment in the gastrointestinal tract.

#### 3.6.2. Surface Properties of BD1

The adhesive capacity of Bifidobacterium, a key trait for colonizing the intestinal mucosa and modulating gut flora, was evaluated. The bacterial adhesion to hydrocarbon (BATH) assay with p-xylene revealed a cell surface hydrophobicity exceeding 30% within 60 min (Figure 7D), indicating pronounced cell surface hydrophobicity. Furthermore, the auto-aggregation ability of BD1 increased steadily with incubation time (Figure 7E). Adhesion density, quantified using ImageJ 1.52i software, was reflected by a root mean square (RMS) value of 120.13 ± 7.80 pixels, corroborating the strain’s biofilm-forming potential (Figure 7F).

## 4. Discussion

*Bifidobacterium animalis* subsp. *lactis*, a probiotic indigenous to the intestinal tract of most mammals including humans, is generally regarded as a strain with superior fermentation traits and health-promoting properties [20]. Owing to these attributes, this bacterium finds widespread application in the food and pharmaceutical industry, solidifying its status as a staple ingredient in probiotic products [21]. The repertoire of *B. animalis* strains suitable for commercial use in food and medicine is still restricted. Consequently, it is imperative to identify and characterize new strains with excellent fermentation performance and proven health benefits to broaden the diversity and application potential of bifidobacterial resources in China. In a previous screen for psychobiotic bifidobacteria, we preliminarily identified a strain of *B. animalis* that demonstrated potential in alleviating neuroinflammation and modulating host mood (data to be published). Although *B. animalis* is generally recognized as safe (GRAS), conventional toxicological assessments alone are insufficient for a comprehensive safety profile. Therefore, it is imperative to employ whole-genome sequencing to re-evaluate the safety of such strains, ensuring their safe application while gaining deeper insights into their functional genomic characteristics.

Based on 16S rRNA gene sequence, the BD1 strain clustered within the *Bifidobacterium animalis* species group, showing 98.83% sequence identity with the reference strain GCF_000260715.1 (Figure 1A). In addition, genomic assembly revealed that the *B. animalis* BD1 strain contains a circular chromosome of 1,935,436 bp, with no plasmids detected (Figure 1B). BD1 exhibits a genome size and chromosome count equivalent to the widely used commercial strain *Bifidobacterium animalis* subsp. *lactis* BB-12 (GCF_000025245.2). Plasmid-free bacterial strains offer distinct advantages in terms of genetic stability, metabolic efficiency, industrial applicability, and biosafety. On the one hand, the absence of plasmids prevents additional genetic fluctuations, enabling long-term stability of strain characteristics, such as the production of specific enzymes and metabolites [22]. On the other hand, since plasmid replication, maintenance, and gene expression (e.g., antibiotic resistance genes or non-essential functional genes) consume substantial energy, nucleotides, amino acids, and other nutrients, plasmid-free strains are relieved of this metabolic burden [22,23]. As a result, they can allocate more resources toward their own growth, reproduction, and targeted metabolic activities, thereby achieving higher metabolic efficiency. Furthermore, many plasmids carry risk genes, such as antibiotic resistance genes and virulence factors, which may spread to other bacteria via horizontal gene transfer [24,25]. The plasmid-free nature of BD1 mitigates this risk, aligning with the safety standards required in food and pharmaceutical applications. Subsequently, functional annotation based on the COG and KEGG databases indicated that the gene functions of *B. animalis* BD1 are primarily enriched in carbohydrate and amino acid transport and metabolism (Figure 2A,C). In fermented foods, microorganisms metabolize carbohydrates and proteins to advance the fermentation process and generate flavor substances, thereby endowing the products with distinct taste and aroma profiles [16,26,27]. Thus, strains equipped with the coding genes or enzymes for carbohydrate and amino acid transport and metabolism are critical in food fermentation. Thus, this finding demonstrates that BD1 possesses the ability to fully utilize carbohydrate and protein substrates through catabolism during fermentation, thereby facilitating the fermentation process and enhancing flavor formation [28,29]. Therefore, BD1 shows potential as a high-quality fermentation strain with promising application prospects in the food industry.

Evaluating strain safety is essential for prospecting new probiotics. Despite the general recognition of *B. animalis* as safe, its precise genomic characterization warrants further investigation. To this end, we conducted a whole-genome-based analysis of virulence, antibiotic resistance, and genomic islands to ensure its safe application. Analysis using the Virulence Factor Database (VFDB) identified 139 genes in the BD1 genome as putative homologs of known virulence factors. However, most of these genes are associated with beneficial bacterial functions, such as adhesion, antimicrobial activity/competitive advantage, stress resistance and membrane biosynthesis [30]. In addition, the sequence similarity between these genes and established viral virulence factors in databases is low, and no related studies have reported virulence in this strain. Together, these lines of evidence indicate that BD1 is not expected to pose a toxic risk, even in the absence of direct phenotypic verification. Moreover, the overuse and misuse of antibiotics have led to the emergence and dissemination of antibiotic-resistant bacteria (ARB) and antibiotic-resistant genes (ARGs), which represent a major public health concern [31,32]. Hence, it is essential to thoroughly analyze the antibiotic resistance profiles and associated genes in bacterial strains. In accordance with the in silico predictions from the whole genome, the antibiotic resistance of strain BD1 was experimentally verified. The microbroth dilution assay revealed that BD1 exhibited resistance solely to tetracycline and mupirocin (Table 2). Notably, these genes are chromosomally encoded and are not flanked by mobile genetic elements (e.g., insertion sequences). The absence of plasmids, coupled with this stable genomic architecture, suggests a minimal risk of horizontal gene transfer. Furthermore, this result indicates that computational predictions should therefore be interpreted with caution; a definitive assessment of key antibiotic resistance traits requires complementary phenotypic verification. The tetracycline resistance in *B. animalis* BD1 is attributed to the *tet*(*W*) gene. This gene is widely distributed among anaerobic intestinal and rumen bacteria and confers resistance to tetracycline [33]. Notably, current studies indicate that the *tet*(*W*) gene encoded by *B. animalis* subsp. *lactis* is part of an ancient resistome and carries a restricted risk of horizontal transfer, supporting a favorable safety profile [34]. Mupirocin is commonly incorporated into selective media for *Bifidobacterium* due to the intrinsic presence of the *ileS* gene, which confers natural resistance in this genus [35]. To date, no evidence suggests that this resistance gene is transmissible to other bacteria via horizontal gene transfer. In contrast to horizontally transferable resistance genes, intrinsic resistance genes (e.g., *tet*(*W*) and *ileS* found in *Bifidobacterium animalis* subsp. *lactis*) generally pose a low risk of horizontal dissemination. The intrinsic resistance is widely recognized as part of the ancient resistome. These genes not only enhance bacterial viability under selective pressure but may also contribute to host intestinal health by enabling persistent colonization during antibiotic therapy and competitively excluding pathogenic organisms. In summary, while BD1 exhibits resistance to tetracycline and mupirocin—mediated by the *tet*(*W*) and *ileS* genes, respectively—both genes are chromosomally encoded and represent common, intrinsic traits among bifidobacteria with a minimal risk of horizontal spread. Therefore, BD1 can be considered safe for use.

Genomic analysis of the BD1 strain identified four genomic islands (GIs), which are proposed to enhance its environmental adaptability and support functional diversification. Among these, GI01 harbors the tetracycline resistance gene *tet*(*W*). However, this island is predominantly composed of genes involved in metabolism and regulation, with no detectable virulence determinants or mobile genetic elements such as insertion sequences [36,37]. As a result, the risk of horizontal gene transfer from GI01 is considered minimal (Figure 3A). Furthermore, these genomic islands exhibit a stable integration into the chromosome, with no evidence of recent horizontal acquisition or mobilization. The absence of flanking attachment sites and integrase genes further reduces the potential for genetic transfer. GI02 displays characteristics resembling a prophage-like element. Nevertheless, screening with the phage prediction tool Phage_Finder did not identify any intact prophage regions in the BD1 genome, suggesting a potential false positive in the initial GI annotation. Therefore, these findings should be interpreted with caution, underscoring certain limitations inherent to genomic prediction (Figure 3B). Moreover, a residual prophage fragment was detected in the reference strain *Bifidobacterium animalis* subsp. *animalis* ATCC 25527 (GCF_000260715.1) but not in BD1, representing a unique genomic trait of this animal-derived subspecies. The remaining genomic islands are associated with core metabolic functions, including genes related to carbohydrate transport and utilization, amino acid biosynthesis, and other essential housekeeping processes. Consequently, mobile genetic elements like genomic islands represent a dynamic interplay of risk and utility. Beyond their potential to disseminate resistance genes, they serve as important drivers of genomic plasticity and adaptive evolution in bacteria. Collectively, the GIs in BD1 can be classified as “metabolic genomic islands,” underscoring the strain’s efficient substrate utilization and robust fermentation performance (Figure 3C,D) [38]. Moreover, given its absence in other reference genomes, this sequence constitutes a unique signature within its genomic context. Furthermore, the BD1 genome contains two insertion sequences. Notably, these sequences are not located on a genomic island and are physically distant from any known antibiotic resistance genes, making it unlikely that they would mediate the dissemination of such resistance determinants under the conditions examined. While insertion sequences were historically classified as potentially harmful genetic elements, contemporary understanding indicates that their activity is modulated by the bacterial regulatory network and environmental signals. This regulated activity can be beneficial, contributing to genetic diversity and enhancing adaptive potential. Moreover, these elements may influence the expression of genes involved in metabolic pathways or host-microbe interactions, thereby potentially affecting functional persistence and maintenance. In summary, genomic analysis confirms that the genomic islands in BD1 do not pose significant biosafety concerns. Instead, they reinforce the strain’s strong metabolic capabilities and support its potential as a high-quality probiotic or industrial starter culture with enhanced environmental adaptability and functional versatility.

Based on the robust metabolic potential revealed by the above analyses, BD1 was identified as a promising starter candidate. We therefore further evaluated its fermentation characteristics, as summarized in Figure 4, Figure 5 and Figure 6. One of the critical attributes underlying the excellent fermentation performance of microbial strains is their capacity for carbon source metabolism. Carbohydrate-active enzymes (CAZymes) represent a major and functionally diverse group of enzymes that play essential roles in this process. These enzymes are categorized into several classes, including glycoside hydrolases, glycosyltransferases, polysaccharide lyases, and carbohydrate esterases. They are responsible for the degradation, modification, and formation of glycosidic bonds, thereby altering the structure of plant-derived polysaccharides and mobilizing stored carbohydrates. For instance, specific saccharification-oriented CAZyme profiles can effectively liquefy starchy plant substrates. Consequently, the repertoire of CAZymes expressed by a strain determines key macroscopic properties—such as texture—of the fermented matrix, modulates the release of metabolites, and directs the synthesis of flavor compounds. These combined effects ultimately contribute to the distinctive sensory characteristics of fermented foods [39,40]. The rich complement of glycosidase and glycosyltransferase genes in BD1 drives its potential ability to finely coordinate sugar metabolism. This coordination directly enhances key fermentation outcomes, including the development of complex flavors, the biosynthesis of functional components, and overall process efficiency, ultimately contributing to superior product quality. Strain BD1 encodes 37 glycoside hydrolase (GH) and 26 glycosyltransferase (GT) family genes, a count comparable to those annotated in other commercial Bifidobacterium genomes. This genomic profile is consistent with a robust capacity for carbohydrate metabolism [41]. KEGG annotation indicates that the metabolic activity of BD1 is concentrated in key pathways including carbohydrate, amino acid, and amino sugar metabolism, which also correlates with its high fermentation proficiency (Figure 2A). The metabolism of these carbohydrates generates organic acids (e.g., lactic and acetic acid), which lower the environmental pH to suppress contaminating microbes, and esters, which contribute to a delicate texture and unique flavor profile in the final product [42,43]. Moreover, the role of microorganisms in different types of fermented meat products has been reported in a large number of studies, and their ability to regulate lipid metabolism can significantly affect the nutritional value and functional characteristics of fermented products [44,45]. The genomic repertoire of BD1 includes genes encoding key enzymes involved in lipid metabolism, indicating its potential for application in fermenting meat and dairy products (Figure 5). Notably, the most prominently enriched pathways are glycerolipid and sphingolipid metabolism (Figure S3). During fermentation, these pathways facilitate lipid hydrolysis, release free fatty acids, improve product texture, enhance nutritional value, and promote the accumulation of flavor precursors [46,47]. Concurrently, the amino sugar and nucleotide sugar metabolism pathway that it participates in supply critical precursors for the synthesis of flavor nucleotides (e.g., IMP and GMP) and potential prebiotics, thereby enhancing the umami taste and the gut-health-promoting value of the fermented food [48,49,50]. In summary, our results position BD1 as a promising starter strain with considerable applicability in the fermentation industry. Future work will focus on phenotypic studies to fully realize this potential.

The previous findings indicating BD1’s potential to alleviate neuroinflammation and regulate host mood (data to be published), and considering the established probiotic status of *B. animalis*, we further characterized its probiotic properties. Experiments confirmed that BD1 exhibits tolerance to gastrointestinal fluids and bile salts, alongside adhesion capability, suggesting its potential to survive gastrointestinal transit, colonize the intestine, and exert beneficial effects (Figure 7). These traits may be attributed to the presence of stress-related genes in the BD1 genome, which enhance its survival under extreme conditions. For instance, we found genes encoding GlsB/YeaQ/YmgE family stress-response membrane proteins and universal stress proteins. These proteins function in sensing environmental stimuli—such as osmotic stress and temperature fluctuations—and participate in regulating membrane lipid composition, cell wall synthesis, membrane permeability, and ion homeostasis, thereby maintaining membrane integrity under stress [51,52]. This gene cluster is also present in *Bifidobacterium animalis* subsp. *animalis* ATCC 25527 (GCF_000260715.1), indicating conservation across these strains. Moreover, BD1 exhibited bile salt tolerance comparable to the commercial strain *Bifidobacterium animalis* BB-12 [53]. Furthermore, it retained a viability of >40% after 4 h of exposure to simulated gastrointestinal fluids. In addition, the genomic islands of BD1 are enriched with genes involved in stress response and regulation, which might collectively improve the strain’s ability to adapt to environmental challenges [54]. Moreover, the genomic repertoire of BD1 showed enrichment in key metabolic pathways for butyrate and propionate biosynthesis (Figure S5). As these short-chain fatty acids are well-established mediators in inflammatory regulation, this genetic background offers a plausible explanation for the strain’s ability to alleviate inflammation [55]. The present study is based on the general recognition of *Bifidobacterium animalis* subsp. *lactis* as a safe probiotic. Accordingly, our analysis relied primarily on whole-genome sequencing predictions, without performing phenotypic validation of some predicted traits, such as fermentation capacity. Future studies will therefore focus on experimental verification using fermentation performance assays. In conclusion, the comprehensive assessment establishes BD1 as a safe probiotic with a favorable safety profile, while simultaneously highlighting its strong potential for industrial fermentation applications.

## 5. Conclusions

Based on comprehensive whole-genome analysis, this study systematically assessed the safety and functional attributes of *B. animalis* BD1. Analysis using the Virulence Factor Database (VFDB) identified homologous genes of potential virulence factors in the strain’s genome; however, most encode proteins associated with biofilm formation and adhesion. Given their low sequence homology to known pathogenic factors, these genes do not pose an actual virulence risk. Antibiotic resistance analysis confirmed the absence of plasmids, indicating a negligible risk of horizontal transfer of resistance genes. Genomic island analysis revealed a functional emphasis on metabolism-related genes, contributing to enhanced metabolic activity and environmental adaptability. Further evaluation of fermentation and probiotic properties demonstrated that BD1 possesses high efficiency in metabolizing carbohydrates and amino acids, along with the potential for host intestinal colonization. These findings are corroborated by our in vivo studies and functional assays (detailed data are to be published). In summary, *B. animalis* BD1 exhibits no detectable toxicity and demonstrates outstanding fermentation performance, probiotic functionality, and environmental resilience, supporting its potential application as a starter culture or probiotic in industrial food fermentation.

## Figures and Tables

**Table 1:** General features of the BD1 genome.

Seq ID	Seq Length (bp)	GC Content (%)	Seq Type	Type	Copy Number
chr	1,935,436	60.49	circular	5S rRNA	3
				16S rRNA	3
				23S rRNA	3
				tRNA	52
				sRNA	9

**Table 2:** Antibiotic resistance test of BD1.

Antibiotics	Breakpoint	*B. animalis* BD1	
		MIC (μg/mL)	Result
Streptomycin	128	64	Sensitivity
Tetracyclines	8	>8	Resistance
Mupirocin	n.r.	>64	Resistance
Rifampicin	1	0.5	Sensitivity

## Data Availability

The original contributions presented in this study are included in the article/Supplementary Materials. Further inquiries can be directed to the corresponding authors.

## Supplementary Materials

The following supporting information can be downloaded at: https://www.mdpi.com/article/10.3390/foods15020316/s1, Supplementary Table S1. Statistical table of the next and third generation quality control data. Supplementary Table S2. Statistical table of genome coverage. Supplementary Table S3. Evaluation table of assembly results of Busco and CheckM. Supplementary Table S4. Prediction of resistance gene of BD1. Supplementary Figure S1. (A) Analysis of read depth versus GC content; (B) Assessment of K-mer frequency distribution for genome assembly evaluation. Supplementary Figure S2. KEGG pathway annotation of carbohydrate metabolism in BD1. The presented pathways include: (A) C5-branched dibasic acid metabolism; (B) Galactose metabolism; (C) Amino sugar and nucleotide sugar metabolism; (D) Starch and sucrose metabolism; (E) Fructose and mannose metabolism; (F) Glyoxylate and dicarboxylate metabolism; (G) Citrate cycle (TCA cycle); (H) Pyruvate metabolism. Enzymes encoded by the BD1 genome are highlighted with red boxes. Supplementary Figure S3. KEGG pathway annotation of lipid metabolism in BD1. The presented pathways include: (A) Glycerolipid metabolism; (B) Glycerophospholipid metabolism; (C) Sphingolipid metabolism. Enzymes encoded by the BD1 genome are highlighted with red boxes. Supplementary Figure S4. KEGG pathway annotation of amino acid metabolism in BD1. The presented pathways include: (A) Cysteine and methionine metabolism; (B) Glycine, serine and threonine metabolism; (C) Alanine, aspartate and glutamate metabolism. Enzymes encoded by the BD1 genome are highlighted with red boxes. Supplementary Figure S5. KEGG pathway annotation of propionate (A) and butyrate (B) metabolism in BD1. Enzymes encoded by the BD1 genome are highlighted with red boxes.

## Abbreviations

The following abbreviations are used in this manuscript:

VFDB	Virulence factor database
KEGG	Kyoto Encyclopedia of Genes and Genomes
GO	Gene Ontology
COG	The Clusters of Orthologous Genes
CAZymes	Carbohydrate-active enzymes
RMS	Root mean square
GDMCC	Guangdong microbial culture collection center
MRS	Man–Rogosa–Sharpe
CDs	Coding sequences
SD	Standard deviation
ANOVA	Analysis of variance
*Hbl*	Hemolysin BL
*Nhe*	Non-hemolytic enterotoxin
*Ces*	Cereulide
MICs	Minimum inhibitory concentrations
GIs	Genomic islands
BATH	Bacterial adhesion to hydrocarbon
GRAS	Generally Recognized as Safe
ARB	Antibiotic-resistant bacteria
ARGs	Antibiotic-resistant genes
