# Supplementary material for: Genome-Based Evaluation of Safety and Probiotic Traits in Infant Feces-Sourced Bifidobacterium animalis subsp. lactis BD1
Source: Foods. 2026 Jan 15;15(2):316. doi: 10.3390/foods15020316 (PMC12841243; doi:10.3390/foods15020316)
Supplement: Supplementary file 1 [file foods-15-00316-s001.zip › foods-4070797-supplementary.pdf]

Tables

Table S1. Statistical table of the next and third generation quality control data.

Next generation sequencing		Third generation sequencing	
Sample Name	BD1	Sample Name	BD1
Insert Size (bp)	474	Total Reads No.	10236
Read Len (bp)	151	Total Bases (bp)	104956032
Raw Pair Reads	10117844	Largest (bp)	22866
Raw Bases (bp)	1527794444	Average Len (bp)	10253.62
Raw Q20 (%)	98.6704	Reads N50 (bp)	10301
Raw Q30 (%)	94.922	/	/
Clean Pair Reads	9945456	/	/
Clean Bases (bp)	1495228090	/	/
Clean Q20 (%)	99.1549	/	/
Clean Q30 (%)	95.7428	/	/

Table S2. Statistical table of genome coverage.

Sample name	Genome Size (bp)	Coverage (%) base on K-mer Analysis	Coverage (%) base on Reads Mapping (Next generation sequencing)	Coverage (%) base on Reads Mapping (Third generation sequencing)
BD1	1935436	100.671843	100	100

Table S3. Evaluation table of assembly results of Busco and CheckM.

CheckM		Busco	
Sample Name	BD1	Sample Name	BD1
Completeness (%)	100	Complete (%)	96
Contamination (%)	0	Complete and single-copy (%)	96
Strain heterogeneity (%)	0	Complete and duplicated (%)	0
/	/	Fragmented (%)	0
/	/	Missing (%)	4

Table S4. Prediction of resistance gene of BD1.

Gene ID	Location	Sample Name	ARO Name
gene0614	Chromosome	BD1	Mycobacterium tuberculosis rpsL mutations conferring resistance to Streptomycin
gene0961	Chromosome	BD1	tet (W)
gene1292	Chromosome	BD1	Bifidobacterium bifidum ileS conferring resistance to mupirocin
gene1345	Chromosome	BD1	Bifidobacterium adolescentis rpoB mutants conferring resistance to rifampicin

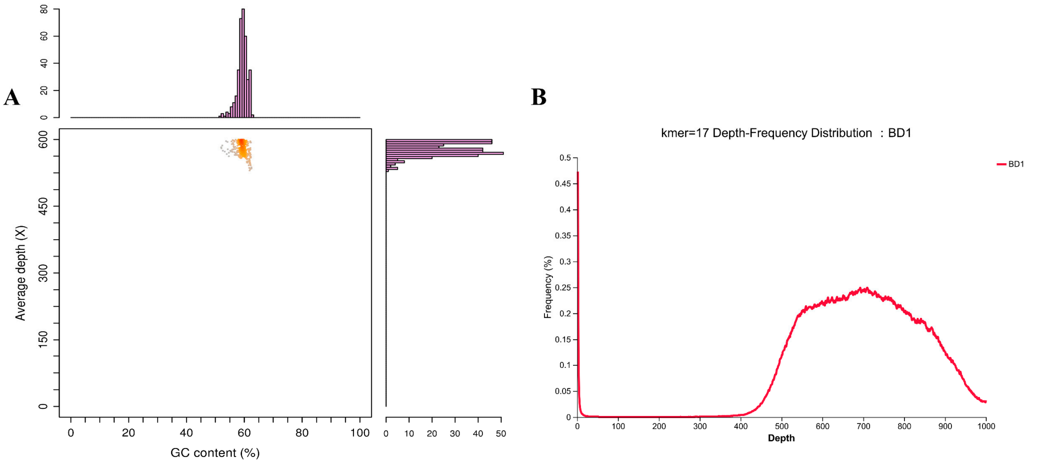

Figure S1. (A) Analysis of read depth versus GC content; (B) Assessment of K-mer frequency distribution for genome assembly evaluation.

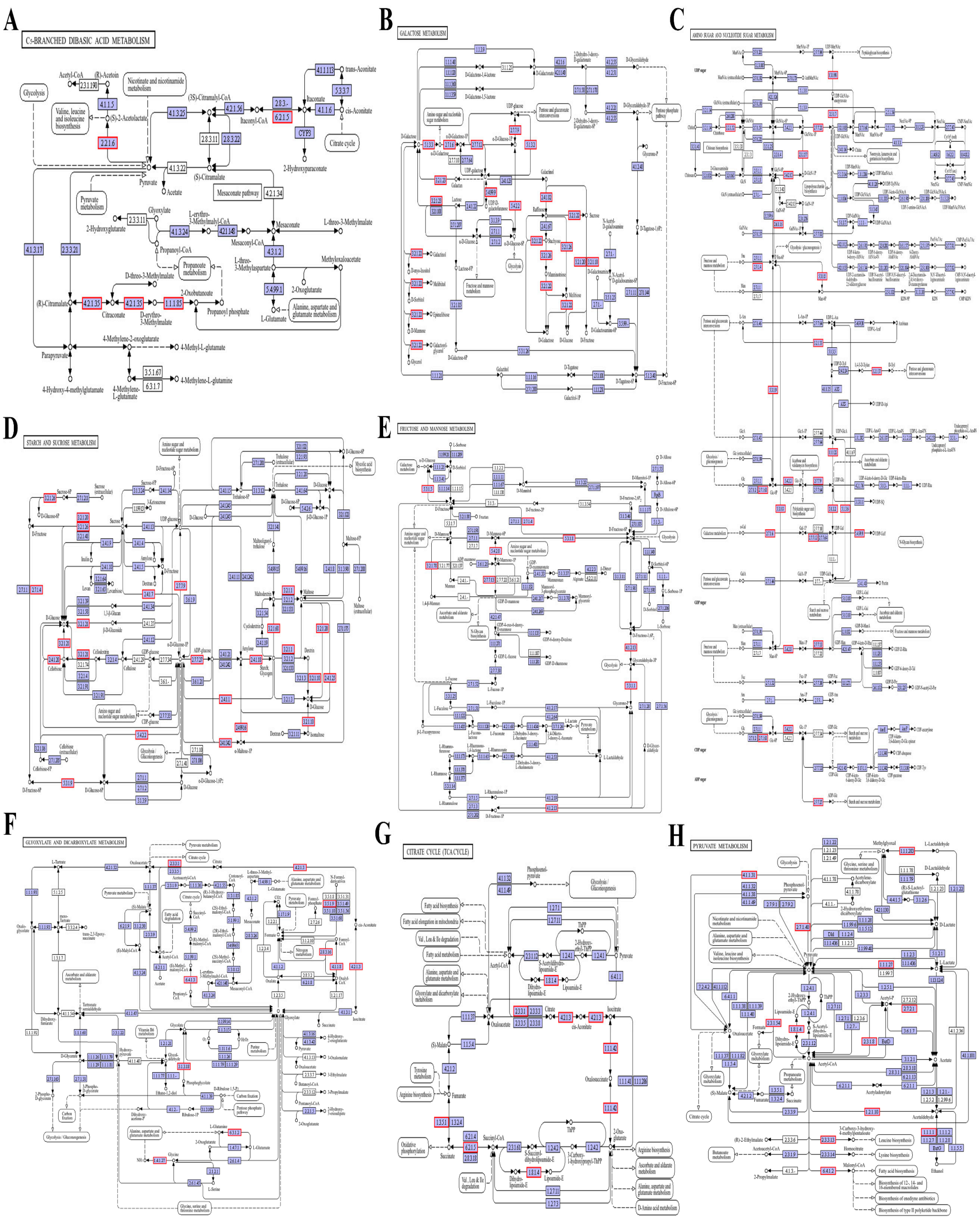

Figure S2. KEGG pathway annotation of carbohydrate metabolism in BD1. The presented pathways include: (A) C5-branched dibasic acid metabolism; (B) Galactose metabolism; (C) Amino sugar and nucleotide sugar metabolism; (D) Starch and sucrose metabolism; (E) Fructose and mannose metabolism; (F) Glyoxylate and dicarboxylate metabolism; (G) Citrate cycle (TCA cycle); (H) Pyruvate metabolism. Enzymes encoded by the BD1 genome are highlighted with red boxes.

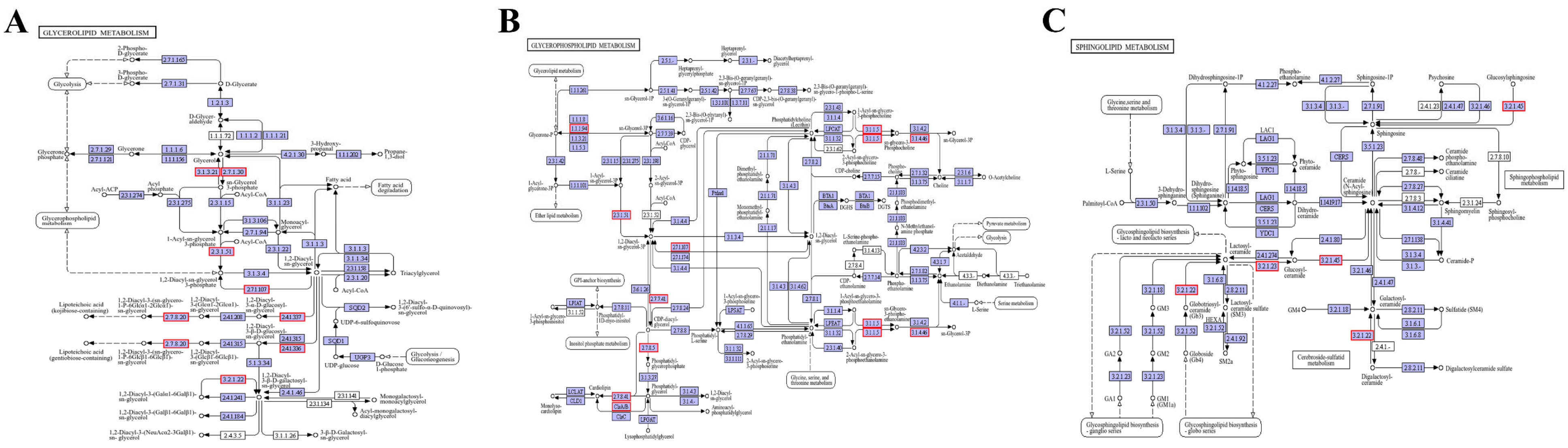

Figure S3. KEGG pathway annotation of lipid metabolism in BD1. The presented pathways include: (A) Glycerolipid metabolism; (B) Glycerophospholipid metabolism; (C) Sphingolipid metabolism. Enzymes encoded by the BD1 genome are highlighted with red boxes.

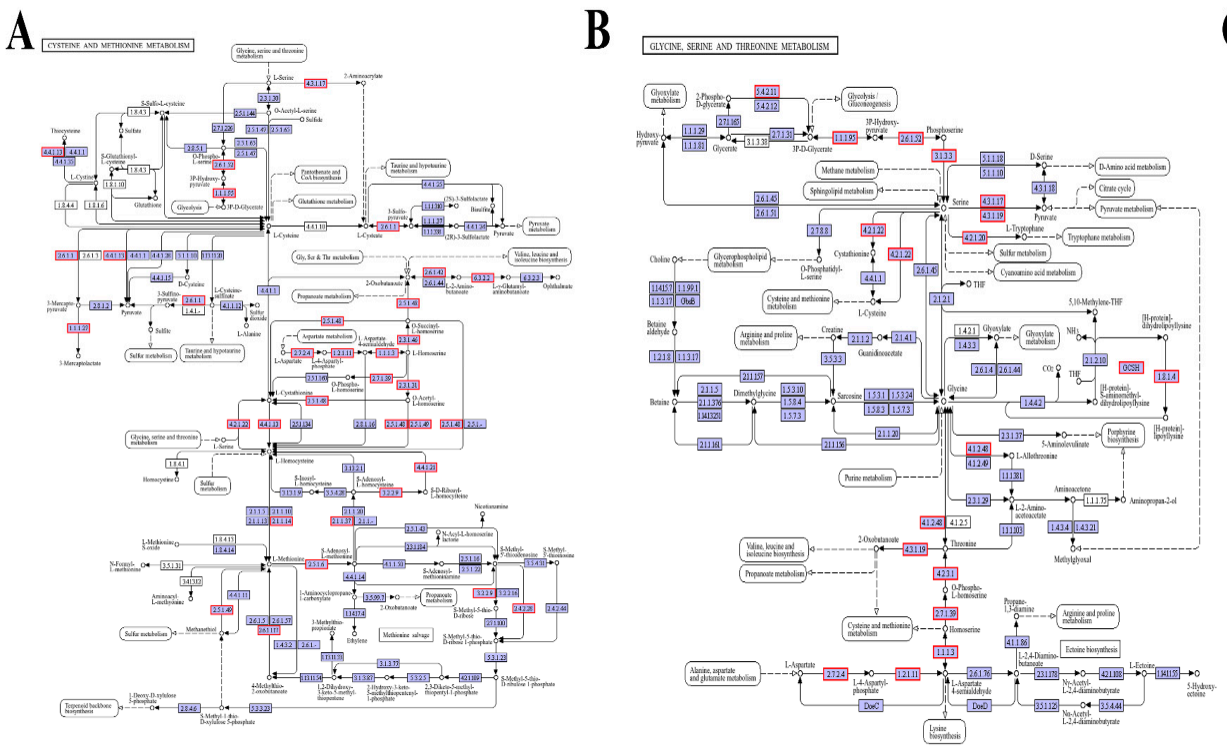

Figure S4. KEGG pathway annotation of amino acid metabolism in BD1. The presented pathways include: (A) Cysteine and methionine metabolism; (B) Glycine, serine and threonine metabolism; (C) Alanine, aspartate and glutamate metabolism. Enzymes encoded by the BD1 genome are highlighted with red boxes.

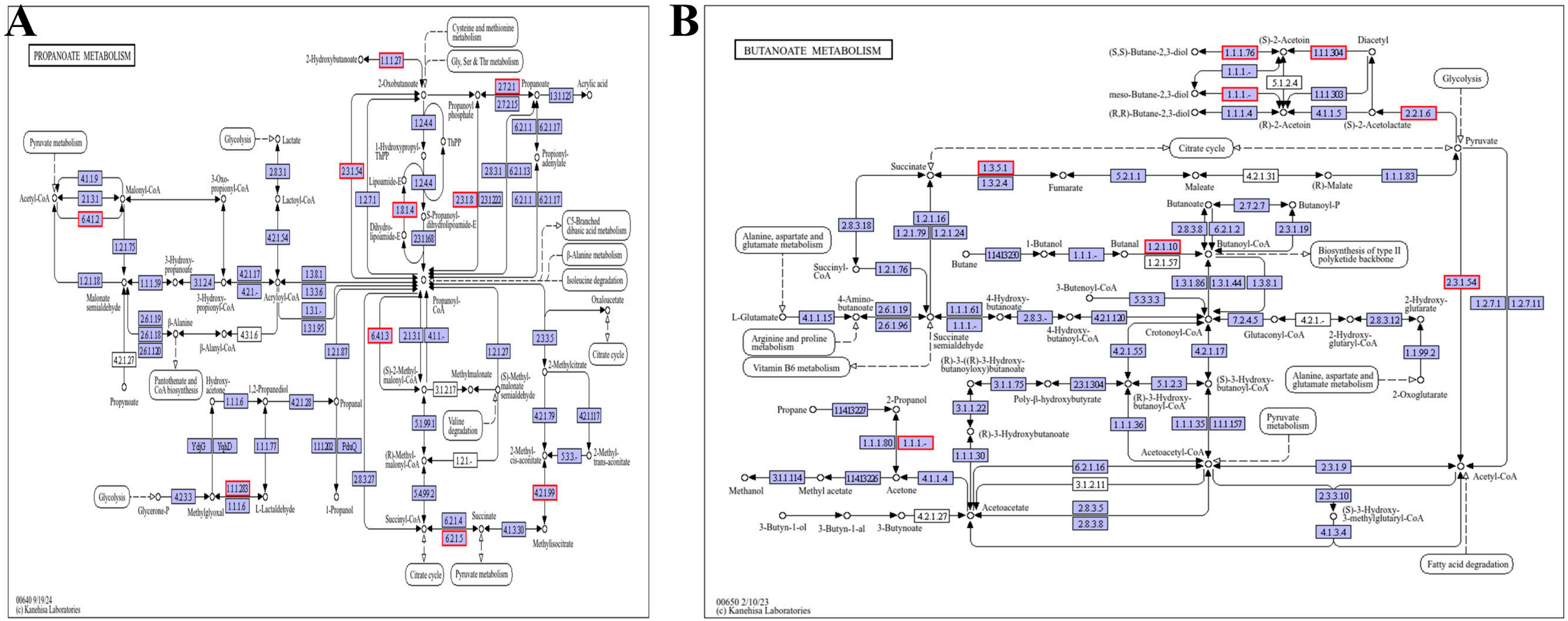

Figure S5. KEGG pathway annotation of propionate (A) and butyrate (B) metabolism in BD1. Enzymes encoded by the BD1 genome are highlighted with red boxes.
